# Can RENAL Nephrometry Scores Predict Perioperative Outcomes in Patients Undergoing Partial Nephrectomy?

**DOI:** 10.7759/cureus.86033

**Published:** 2025-06-15

**Authors:** Chithra Sugathan Sheela, Renu Thomas, K Sasidharan

**Affiliations:** 1 Urology, Queen Elizabeth Hospital Birmingham, Birmingham, GBR; 2 Urology, KIMSHEALTH, Thiruvananthapuram, IND

**Keywords:** partial nephrectomy, peri-operative outcomes, renal nephrometry score, small renal mass, s: renal cell carcinoma

## Abstract

Objective

Preoperative imaging-based scoring systems help choose the intervention of choice and can help predict postoperative complications in patients undergoing surgery for small renal masses. The study aims to evaluate the utility of RENAL Nephrometry Score (RNS) in predicting intraoperative ischemia times, estimated intraoperative blood loss, and postoperative complications in patients undergoing partial nephrectomy.

Methods

A total of 40 consecutive patients undergoing partial nephrectomy at a tertiary care hospital in South India were recruited into a prospective observational study. The preoperative imaging-based RENAL scores were obtained, and various intraoperative (ischemia times, blood loss) and postoperative variables (duration of hospital stay, change in renal function, and postoperative complications) were compared.

Results

A total of 80% of patients had low RENAL scores, while 20% had high scores. There was a statistically significant association between RENAL scores and intraoperative ischemia times(p=0.024) and tumor size(p=0.008). Other variables like blood loss, postoperative complications, duration of hospital stay, and change in renal function did not show any association with RENAL scores.

Conclusions

The RNS is a useful tool in predicting intraoperative ischemia times in patients undergoing partial nephrectomy for small renal masses. It can also be useful to predict tumor size in the final histopathological assessment of renal cell carcinoma. Comparative studies incorporating larger numbers of patients are required to establish statistically significant associations between RNS and the secondary outcomes proposed by this study.

## Introduction

Renal cell carcinoma is the most fatal of the commonly occurring urological malignancies. Traditional recommendations advocated radical nephrectomy for solid renal masses, with partial nephrectomy reserved for patients with solitary kidney or bilateral renal masses. During the last decade, partial nephrectomy has emerged as a standard treatment for small renal masses, offering oncologic control equivalent to radical nephrectomy with preservation of renal function and evidence for equivalent survival [1]. The selection of patients for the nephron-sparing approach to managing renal masses has been greatly subjective. It has depended on patient comorbidities, location and size of mass, surgeon experience, and available facilities, as well as patient choice.

The RENAL Nephrometry Score (RNS) was developed by Kutikov and Uzzo [2] to standardise the assessment of anatomical features of renal tumours. The scoring system is based on the five most reproducible features that characterize the anatomy of a solid renal mass: R: Radius‑scores tumor size as maximal diameter; E: Exophytic/endophytic properties of the tumour; N: Nearness of the deepest portion of the tumour to the collecting system or renal sinus; A: Anterior (a)/posterior (p) descriptor; L: Location relative to the polar line [2].

All components except for the (A) descriptor are scored on a 1‑, 2‑, or 3‑point scale. The (A) describes the principal mass location to the coronal plane of the kidney. The additional suffix “h” is used to designate a hilar location of the tumour (abutting the main renal artery or vein) [2]. Patients are divided into three groups depending on the complexity scores (low complexity score 4-6, moderate complexity score 7-9, and high complexity score 10 or more) [2].

The RNS thus helps urologists with the possible technical difficulty during partial nephrectomy for a given mass and has been correlated with ischemia time, operation time, blood loss, complications, and the likelihood of conversion from partial nephrectomy to radical nephrectomy [2].

## Materials and methods

This prospective study was done at KIMSHEALTH, Thiruvananthapuram, a tertiary care hospital in South India.

After obtaining clearance from the institutional ethics committee, 40 consecutive patients undergoing partial nephrectomy for small renal masses were recruited into the study between June 2020 and December 2021.

All necessary preoperative investigations and radiological assessments were done per the hospital protocol. Written informed consent was obtained after explaining the purpose of this study and after providing the patient information sheet. The investigator evaluated all the consenting patients, and detailed history-taking and clinical examination were conducted. Contrast CT images and reports were evaluated, and the RENAL nephrometry score was arrived at. Subsequently, patients were classified into two groups for the purpose of the study: those with scores up to 7 were the ‘low score group’, and those with scores above 7 were the ‘high score group’.

During the surgical procedure, intraoperative variables were recorded. These include ischemia time (duration of vessel clamping), use of hypothermia, estimated blood loss, and requirement of re-clamping. Postoperatively, blood investigations were done on the first post-operative day, as per hospital protocol, which was investigated. Patients were followed up till discharge, to look for the development of complications, and subsequently, until the first post op OPD visit. Surgical complications were classified using the Clavien-Dindo classification system to ensure standardisation [3]. 

Histopathology reports were followed up to obtain variables such as tumor size, TNM stage, tumor histology, margins, and nuclear grade. The variables so obtained were compared between the two groups to assess whether the nephrometry score can be used to predict perioperative outcomes in patients undergoing partial nephrectomy. The primary objective was to assess the utility of the RENAL nephrometry scores in predicting intraoperative ischemia times. Secondary objectives included the usefulness of the scores in predicting perioperative outcomes, including intraoperative blood loss, fall in serum creatinine, post-operative complications, and final histopathology reports. 

The data collected was tabulated using Microsoft Excel (Microsoft Corp., Redmond, WA). All variables were summarized using frequencies and percentages. The chi-square test was used to find the association between categorical variables. For all statistical interpretations, p<0.05 was considered the threshold for statistical significance. Statistical analysis was performed with SPSS version 20.0 (IBM Corp., Armonk, NY).

## Results

Our study population's demographic distribution showed that most patients who underwent partial nephrectomy for renal masses were more than 40 years old, with 40% (n=16) between 40 and 60, and 35% (n=14) above 60. The study population also showed a male preponderance (72.5%, n=29). Around 70% of patients (n=28) had a BMI over 25, and 40% (n=16) were hypertensive. Most of the renal masses were incidentally detected (82%, n=33). 

**Figure 1:**
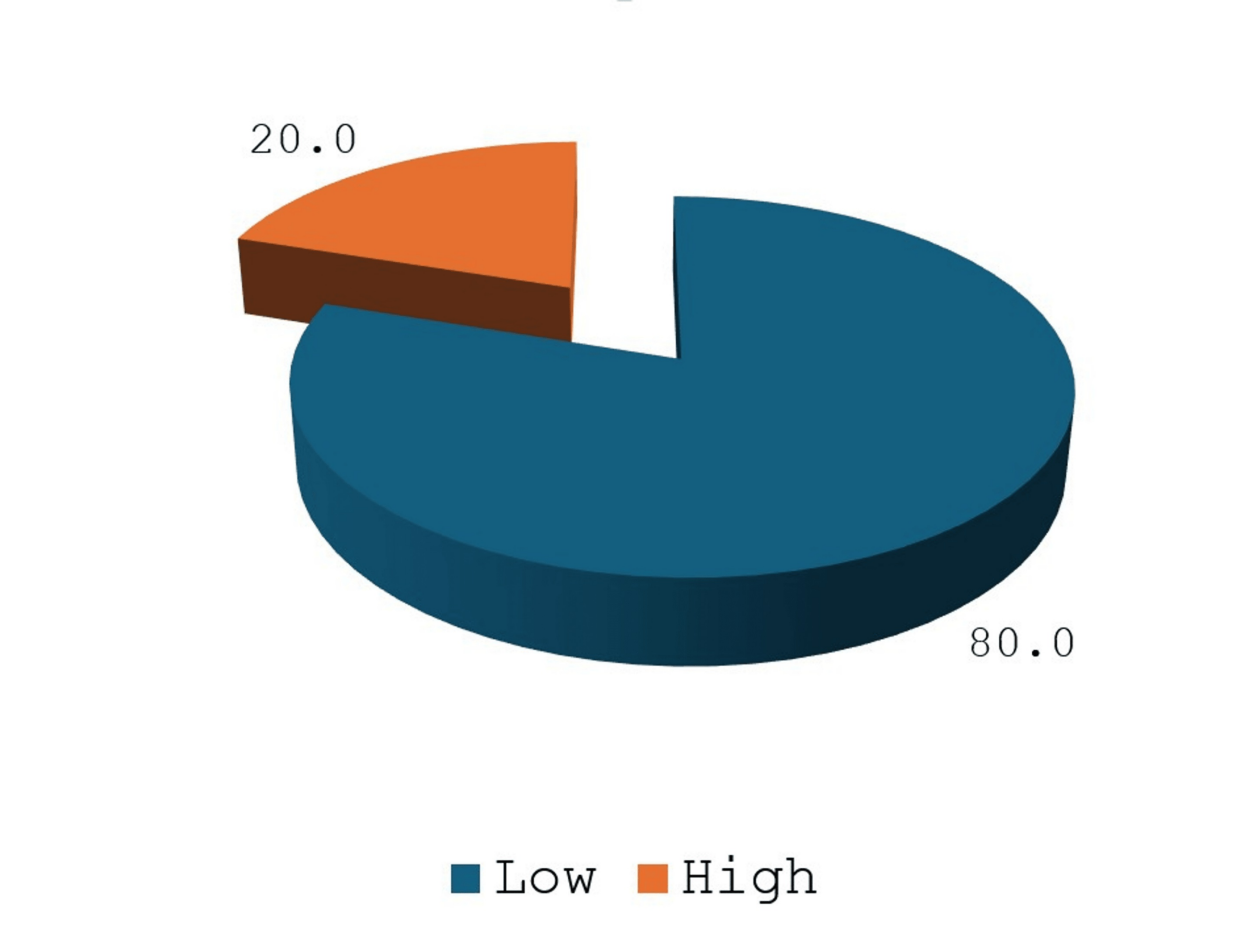
Percentage distribution of the sample according to low/high RENAL score The pie chart shows the distribution of the study population (expressed as percentages) based on high or low RENAL scores. For the study, scores up to 7 were considered low, and scores above seven were considered high. A total of 32 patients (80%) had low scores, and eight patients (20%) had high RENAL scores.

Most patients (80%, n=32) had low RENAL scores, while only 20% (n=8) had high scores, which were arbitrarily decided to be up to 7 and above 7 for our study (Figure 1). In agreement with this, the imaging-based TNM stage of most of the study population was T1aN0 (67.5%, n=27), followed by T1bN0 (27.5%, n=11) and a small proportion with T2N0 (5%, n=2) lesions.

Ischemia times were mainly between 10 and 30 minutes, and almost 50% (n=19) of cases were operated on without the use of intraoperative hypothermia. Only one patient required reclamping intraoperatively; however, the blood loss was not significant in any of the patients studied. 

Postoperative hemoglobin fall was used as a surrogate for intraoperative blood loss. Although 90% (n=36) of the study population showed a fall in hemoglobin in the immediate postoperative assessment, only 41% (n=15) had a fall >1g/dl. Postoperative rise in creatinine was also assessed. Though 85%(n=34) of the population studied showed a rise in serum creatinine, only one patient showed a rise of >1mg/dl. 

Only five patients (12.5%) had prolonged hospital stays lasting more than a week. Around 55% of the study population did not show any post-operative complications (n=22), and 7.5% (n=3) each had Clavien-Dindo grade 3a and above complications. Only one patient in the study population had multiorgan dysfunction and an ICU stay, and one patient required intervention under general anaesthesia (nephrectomy secondary to bleeding unresponsive to Radiological intervention).

Upon reviewing the histopathology results, most patients had clear cell RCC (67.5%, n=27), and 17.5% had benign histology- oncocytoma (n=2) and angiomyolipoma (n=5). Other RCC variants, like papillary and chromophobe variants, were present, though only in smaller numbers (papillary RCC 2/40, and chromophobe RCC 1/40). 

**Figure 2:**
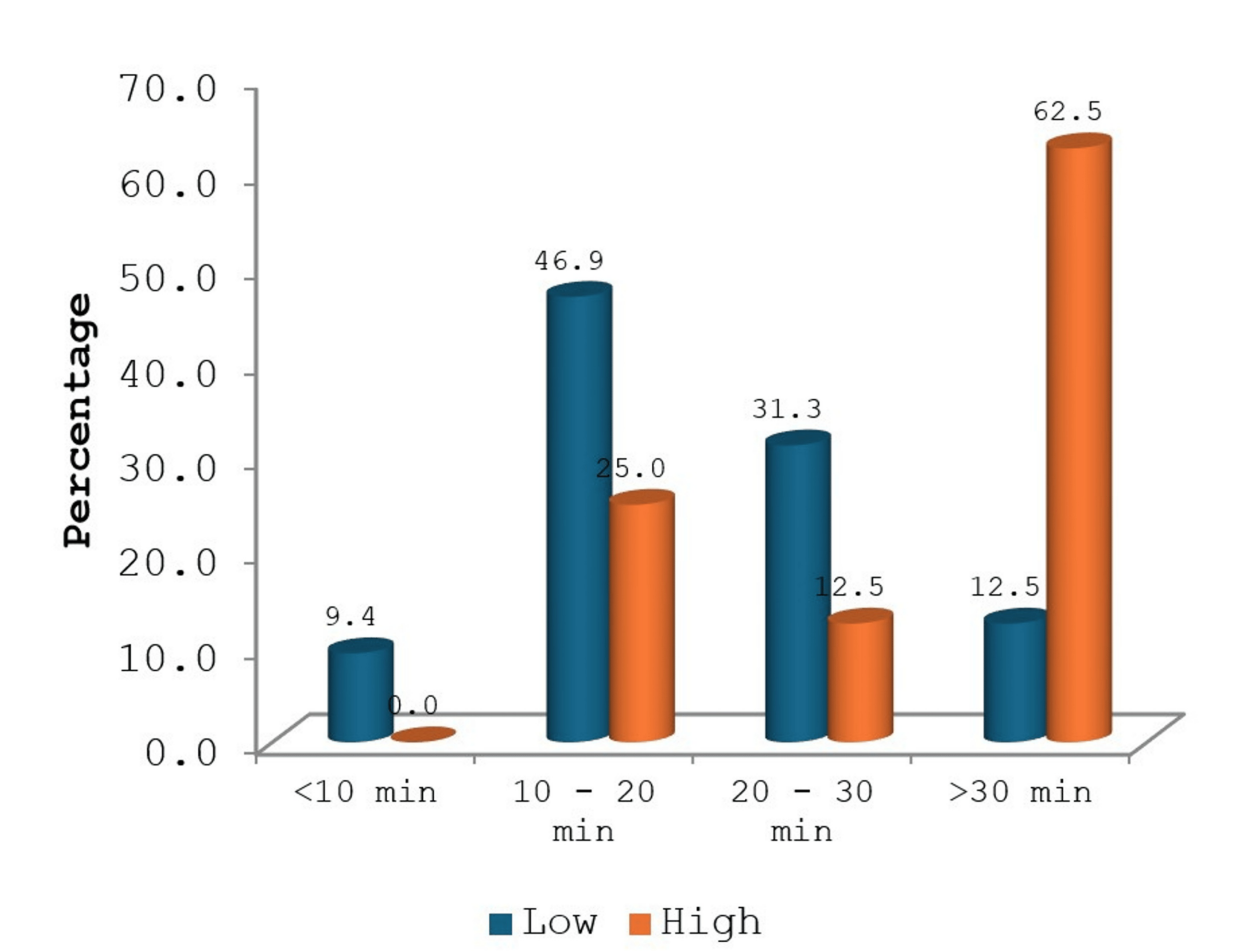
Comparison of ischemia times based on RENAL score The figure shows a comparison of intraoperative ischemia times with RENAL scores. Longest ischemia times were noted for patients with higher RENAL scores and this reached statistical significance. (p<0.05)

We compared the intraoperative ischemia times with RENAL scores and found that the patients with higher scores also had longer ischemia times (Figure 2). This finding was statistically significant (p=0.024). Around 80% of the patients with low RENAL scores had a tumor size of less than 4 cm, while 70% of the high-scoring patients had a tumor size of more than 4 cm. This was found to be statistically significant as well (p=0.008). The threshold for statistical significance was considered a p-value <0.05.

**Figure 3:**
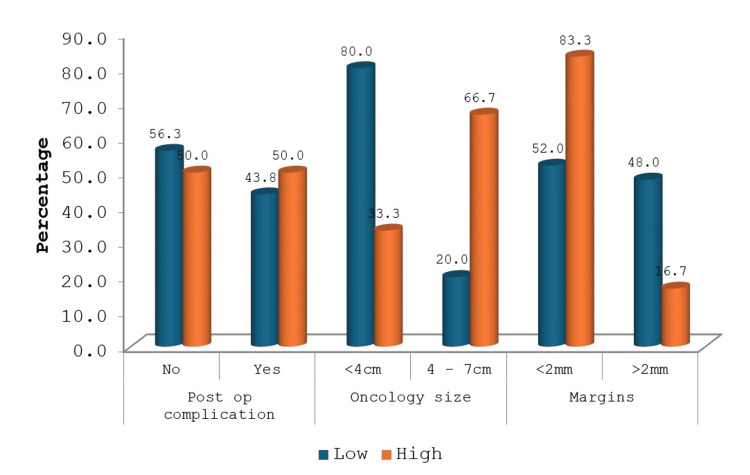
Comparison of selected variables based on RENAL score The figure compares variables like post-operative complications, tumour size and surgical margins with RENAL scores.

When we looked at the post-operative complications, we found that 50% (n=2) of the high-scoring patients developed complications, but these scored 2 or below on the Clavien-Dindo scale. Around 83% (n=5) of patients with high RENAL scores had <2 mm resection margins. These findings were not statistically significant (Figure 3). Intraoperative blood loss and postoperative renal function deterioration did not show any relation to RENAL scores, nor did postoperative hospital stay.

## Discussion

Renal cell carcinoma is more common in males and in the elderly. Obesity and hypertension are known risk factors for the development of renal cancer [1]. With the increasing use of abdominal imaging for a variety of indications, the incidental detection of renal masses has become more common. Complexity scores like the RENAL nephrometry score can aid the clinician in choosing between partial and radical nephrectomy for these patients. Less complex lesions are chosen for partial nephrectomy, and more complex lesions for radical surgery. This process of patient selection is reflected in our study population as well, with only 20% of patients undergoing partial nephrectomy having high scores. 

The most common variant of renal cancers is the clear cell variety [1], which is also reflected in our study population. A total of 56% of the patients had microscopic resection margins below 2mm. Close resection margins or even positive resection margins in patients undergoing partial nephrectomy are no longer considered indications for re-resection. Such patients are followed up as is protocol after surgery. Studies have shown that they are not at increased risk for local recurrence or distant metastasis [4,5]. Nuclear grade, as a measure of the degree of differentiation of the tumor, showed less than 10% of the study population with high nuclear grade lesions.

There was a statistically significant association between intraoperative ischemia times and the complexity of lesions, with higher scoring tumors having longer ischemia times. This finding correlates with a retrospective study conducted in a tertiary care hospital in China. Of the 139 patients studied, those with higher RNS were found to have longer ischemia times [6]. However, an interesting finding is that a retrospective study from Korea, among 98 patients undergoing partial nephrectomy under cold ischemia, did not demonstrate the same relation between ischemia time and RENAL scores [7]. Nearly 50% of our study population was operated on without hypothermia. Whether this has contributed to our findings is unclear.

In most studies done on partial nephrectomy, the majority of patients showed a lower grade of complications and were managed conservatively. This was true even in studies with a definite correlation between RNS and postoperative complications. This is possibly a reflection of the fact that most of these studies were done in centres that have a careful process of patient selection and the surgical expertise needed to perform this procedure safely. A multicentre retrospective study from the Netherlands demonstrated that an RENAL score above 9 can independently predict post-operative complications [8]. Only 20% of our study population had a score >7, and of these, only one had a score above 10. This could possibly have contributed to the absence of a statistically significant relation between RENAL score and post-operative complications. 

Though not statistically significant, most patients with high scores had close margins of resection. This is representative of the practical difficulty of getting wide margins in complex lesions.

This study used postoperative haemoglobin fall as a surrogate for estimating intraoperative blood loss. Most studies on partial nephrectomy have demonstrated a relationship between higher RENAL scores and estimated intraoperative blood loss [6-9]. The smaller number of patients with higher RENAL scores and overall lower complexity of the high-scoring lesions has perhaps resulted in this confounding picture. One patient with a low score had a rise in creatinine above 1mg/dl post-operation, and none of the patients with high scores had such a rise. This finding can be explained by a German study that demonstrated that the Centrality Index was a scoring system better suited to predicting renal function outcomes post partial nephrectomy [10]. However, other studies, for example, the retrospective analysis by Reddy et al. from the UK [11] and the prospective study by Basu et al. from India [9] have demonstrated a correlation between higher RENAL scores and greater postoperative decline in renal function.

The study was carried out at a tertiary centre, so referral bias was inevitable. Around 80% of the study population had low RENAL scores, so the numbers were not comparable. This is probably because of the observational nature of the study. The relatively low complication rate, as per our study, could reflect the secure technique utilized for each of our patients; however, a false negative due to the smaller sample size could have played a contributory role. The operating steps and CT reporting were not standardised, which could also have contributed to the limitations of the study.

## Conclusions

This prospective study in a high-volume tertiary care hospital in South India aimed to assess whether the complexity score assigned to renal mass based on preoperative imaging could predict perioperative outcomes in patients undergoing partial nephrectomy. It established the utility of the RENAL nephrometry score in predicting ischemia times and final tumour size. This can guide the operating surgeon to plan the use of intraoperative hypothermia and counsel patients appropriately before surgery. Though statistical significance was not achieved in the secondary outcomes of interest, including postoperative complications, this study highlights the need for improved study design to more accurately evaluate these associations.
